# Use of concomitant inhaled corticosteroids: pooled data from two phase III studies of aclidinium plus formoterol in COPD

**DOI:** 10.1038/s41533-016-0009-3

**Published:** 2017-02-24

**Authors:** Anthony D’Urzo, Dave Singh, Esther Garcia Gil

**Affiliations:** 1 Department of Family and Community Medicine, Faculty of Medicine, University of Toronto, Toronto, Canada; 2 The University of Manchester, Medicines Evaluation Unit, University Hospital of South Manchester Foundation Trust, Centre for Respiratory and Allergy Medicine, Manchester, UK; 3 R&D Centre, AstraZeneca, Barcelona, Spain

## Abstract

Bronchodilator therapy is the backbone of the management of chronic obstructive pulmonary disease. In some patients, inhaled corticosteroids can be prescribed in combination with bronchodilators. Through a subgroup analysis of pooled data from two large phase III clinical trials of bronchodilator therapy according to concomitant inhaled corticosteroid use (user vs. non-user), we sought to evaluate the clinical benefit of adding inhaled corticosteroids to dual bronchodilator therapy in chronic obstructive pulmonary disease. The primary focus of this analysis of pooled data from the phase III ACLIFORM and AUGMENT studies was to evaluate the efficacy of aclidinium/formoterol on lung function stratified by inhaled corticosteroid use. We found that lung-function end points were significantly improved regardless of concomitant inhaled corticosteroid use among patients treated with the dual bronchodilator aclidinium/formoterol 400/12 µg twice daily compared with placebo and both monotherapies. Together with the previously reported observations that aclidinium/formoterol 400/12 µg reduces exacerbations vs. placebo in inhaled corticosteroid users and improves dyspnoea compared to monotherapy in inhaled corticosteroid non-users, these data suggest that both groups achieve lung function improvements, which translates to different clinical benefits depending on whether or not a patient is receiving concomitant inhaled corticosteroids.

## Chronic lung disease: ‘Triple’ therapy could prove beneficial

A dual bronchodilator therapy taken together with corticosteroid inhalers may benefit patients with severe chronic lung disease. Bronchodilator drugs relax the lungs and widen airways in patients with chronic obstructive pulmonary disease (COPD). While recent studies have shown that a dual bronchodilator therapy containing aclidinium and formoterol significantly improves lung function in COPD, little is known about combining the dual therapy with inhaled corticosteroids (ICSs). Anthony D’Urzo at the University of Toronto, Canada, and co-workers analysed data from 3394 patients with COPD undergoing dual therapy trials. Of these, 1180 were already taking ICSs. The team compared symptoms in the ICS group with those not taking ICSs. The dual therapy improved lung function across both groups regardless of ICS use, though patients gained different clinical benefits depending on ICS use and disease severity.

## Introduction

The combination of aclidinium bromide (400 μg), a long-acting muscarinic antagonist (LAMA), with the long-acting beta-2 agonist (LABA) formoterol fumarate (12 μg) administered twice daily is currently approved for the treatment of chronic obstructive pulmonary disease (COPD) in the European Union and in other parts of the world, including Australia and Canada.^1–3^ Two large phase III, randomised, double-blind, 24-week trials—ACLIFORM (NCT01462942) and AUGMENT (NCT01437397)—in patients with COPD showed significant improvements in lung function with this dual bronchodilator combination compared with placebo and monotherapies.^4, 5^ A pooled, secondary analysis of these studies also demonstrated a significant improvement in symptoms with this dual bronchodilator combination compared to placebo and monotherapies.^6^

Concomitant use of inhaled corticosteroids (ICSs) was permitted in ACLIFORM and AUGMENT, as it was considered safer to allow patients to continue on this treatment. This was particularly important for the placebo arm who received no long-acting bronchodilator maintenance therapy. The continuation of previous ICS use is a common feature of clinical studies of LABA+LAMA combination therapies^7–9^ and, in line with current guidelines, patients with COPD with an elevated risk for exacerbation can be prescribed ICS in combination with bronchodilators.^10^ However, in clinical practice many patients will use dual bronchodilator combinations without an ICS, including as a step up from long-acting bronchodilator monotherapy. Consequently, clinical trials such as ACLIFORM and AUGMENT are composed of two subgroups according to ICS use, of which ICS non-users could be considered to be the more relevant target population for dual bronchodilator combinations.

In ACLIFORM and AUGMENT, patients were not randomised to receive ICS and the studies were not designed to assess ICS use. However, the presence of an ICS-user population means that some patients were treated with ‘triple therapy’ (ICS+LAMA+LABA) and could be compared to patients receiving ‘dual therapy’ containing an ICS (ICS+LABA or ICS+LAMA).

The recently published pooled, secondary analysis of ACLIFORM and AUGMENT, stratified by concomitant ICS use, reported that, compared with placebo, aclidinium/formoterol 400/12 µg improved dyspnoea regardless of concomitant ICS use.^6^ The same analysis demonstrated that the rate of exacerbations was much higher among ICS users than ICS non-users, and that aclidinium/formoterol 400/12 µg reduced the rate of exacerbations compared with placebo in those patients using concomitant ICS.^6^

The efficacy of aclidinium/formoterol on lung function, stratified by ICS use, has not yet been reported for ACLIFORM and AUGMENT and is the primary focus of this pooled, secondary analysis, since providing optimal bronchodilation is pivotal in the management of COPD. Here, we report results for the co-primary efficacy end points, change from baseline in morning pre-dose (trough) and morning 1-h post-dose forced expiratory volume in 1 s (FEV_1_) at Week 24.

## Results

Of 3394 patients analysed (Table 1), 1180 (34.8%) were ICS users and 2214 (65.2%) were non-ICS users. The proportion of patients with severe COPD was greater in ICS users vs. non-ICS users at baseline (49.3% and 36.9%, respectively), as was the proportion of patients with at least one exacerbation in the previous 12 months (35% and 26%, respectively) (Table 1). In the ICS subgroup, the most frequently used therapies were fluticasone (45.3%; dose range 100 µg–1 mg/day), budesonide (35.1%; dose range 100 µg–2 mg/day) and beclomethasone (12.3%; 100 µg–2 mg/day) (Table 2).

**Table 1:** Patient demographics in patients with COPD using ICS and those not using ICS

Characteristic	Concomitant ICS use^a^ (*n* = 1180)	No concomitant ICS use (*n* = 2214)
Mean age, years (SD)	64.5 (7.8)	63.0 (8.7)
Sex, male, *n* (%)	695 (58.9)	1358 (61.3)
Current smoker, *n* (%)	502 (42.5)	1174 (53.0)
FEV_1_, *L* (SD)	1.27 (0.47)	1.45 (0.54)
Post-bronchodilator FEV_1_, % predicted (SD)	45.70 (13.58)	49.80 (14.26)
Post-bronchodilator reversibility, *n* (%)	439 (37.3)	872 (39.5)
COPD severity,^b^ *n* (%)		
I (mild)	1 (0.1)	4 (0.2)
II (moderate)	597 (50.7)	1390 (62.9)
III (severe)	578 (49.1)	804 (36.4)
IV (very severe)	2 (0.2)	12 (0.5)
Mean number of exacerbations in previous 12 months (SD)	0.5 (0.8)	0.4 (0.8)
Number of exacerbations in previous 12 months (%)		
0	767 (65.0)	1639 (74.0)
1	296 (25.1)	392 (17.7)
≥2	117 (9.9)	183 (8.3)

*COPD* chronic obstructive pulmonary disease, *FEV*_1_ forced expiratory volume in 1s, *FVC* forced vital capacity, *ICS* inhaled corticosteroid, *SD* standard deviation

^a^Concomitant ICS use was defined as any ICS used at baseline (in the 15 days prior to study start), and continued throughout the treatment period

^b^COPD severity: I, FEV_1_ ≥80% predicted and FEV_1_/FVC <0.70; II, FEV_1_ 50–80% predicted and FEV_1_/FVC <0.70; III, FEV_1_ 30–50% predicted and FEV_1_/FVC <0.70; IV, FEV_1_<30% predicted and FEV_1_/FVC <0.70

**Table 2:** Concomitant ICS used by the ICS group

ICS	Percentage
Fluticasone	45.3
Budesonide	35.1
Beclomethasone	12.3
Mometasone	4.2
Ciclesonide	3.1

*ICS* inhaled corticosteroid

### Lung-function measures

#### 1-h post-dose FEV_1_

**Fig. 1:**
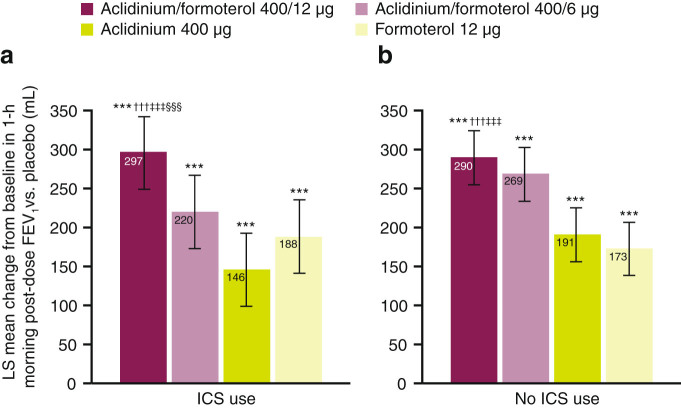
LS mean change from baseline in 1-h morning post-dose FEV_1_ vs. placebo at week 24 in ICS users **a** and non-ICS users **b** (pooled ITT population). ***P < 0.001 vs. placebo; ^†††^ *P* < 0.001 vs. aclidinium 400 μg; ^‡‡‡^ *P* < 0.001 vs. formoterol 12 μg; ^§§§^ *P* < 0.001 vs. aclidinium/formoterol 400/6 μg. Analyses based on the mixed model for repeated measures: treatment effects and treatment comparisons. Error bars represent 95% confidence intervals. Patient numbers for the placebo groups were: for patients using ICS, *n* = 137, for patients not using ICS, *n* = 251. *FEV*_1_ forced expiratory volume in 1 s, *ICS* inhaled corticosteroid, *ITT* intent-to-treat, *LS* least squares, *mL* millilitre

At week 24, improvements in 1-h post-dose FEV_1_ were observed for both doses of aclidinium/formoterol vs. placebo irrespective of ICS use (*P* < 0.001 for all comparisons; Fig. 1). With aclidinium/formoterol 400/12 µg, the licensed dose, the improvements vs. placebo were 297 mL for ICS users and 290 mL for non-ICS users (*P* < 0.001 for both comparisons; Fig. 1). In ICS users, aclidinium/formoterol 400/12 µg caused an increase in 1-h post-dose FEV_1_ of 108 mL vs. formoterol (*P* < 0.001) and 151 mL (*P* < 0.001) vs. aclidinium. A similar pattern of improvement was observed with aclidinium/formoterol 400/12 µg in non-ICS users: 117 mL vs. formoterol and 99 mL vs. aclidinium (both *P* < 0.001). A direct comparison between ICS users and non-users found that there were no significant differences in 1-h post-dose FEV_1_ improvements with ICS use for any treatment group (Table 3).

**Table 3:** Lung function end points: ICS users vs. non-ICS users

	Treatment difference ICS vs. non-ICS, mL (SE)	*P*-value
*LS mean change from baseline in 1-h post-dose FEV*_*1*_ *at week 24*		
Placebo	19 (23)	0.405
Aclidinium/formoterol 400/12 µg	27 (19)	0.142
Aclidinium/formoterol 400/6 µg	−32 (19)	0.096
Aclidinium 400 µg	−33 (19)	0.082
Formoterol 12 µg	35 (19)	0.063
*LS mean change from baseline in trough FEV*_*1*_ *at week 24*		
Placebo	2 (22)	0.922
Aclidinium/formoterol 400/12 µg	15 (18)	0.410
Aclidinium/formoterol 400/6 µg	−28 (18)	0.129
Aclidinium 400 µg	−32 (18)	0.081
Formoterol 12 µg	9 (18)	0.637

*FEV*_*1*_ forced expiratory volume in 1 s, *ICS* inhaled corticosteroid, *LS* least squares, *mL* millilitre, *SE* standard error

#### Trough FEV_1_

**Fig. 2:**
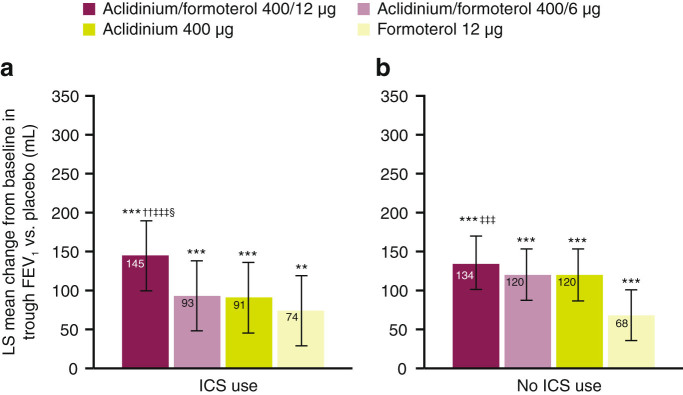
LS mean change from baseline in trough FEV_1_ vs. placebo at week 24 in ICS users **a** and non-ICS users **b** (pooled ITT population) **P < 0.01 vs. placebo; ***P < 0.001 vs. placebo; ^††^ *P* < 0.01 vs. aclidinium 400 μg; ^‡‡‡^ *P* < 0.001 vs. formoterol 12 μg; ^§^ *P* < 0.05 vs. aclidinium/formoterol 400/6 μg. Analyses based on the mixed model for repeated measures: treatment effects and treatment comparisons. Error bars represent 95% confidence intervals. Patient numbers for the placebo groups were: for patients using ICS, *n* = 137, for patients not using ICS, *n* = 254. *FEV*_1_ forced expiratory volume in 1 s, *ICS* inhaled corticosteroid, *ITT* intent-to-treat, *LS* least squares, *mL* millilitre

All active treatments improved trough FEV_1_ compared with placebo at week 24, irrespective of ICS use (all *P* < 0.05; Fig. 2). Treatment with aclidinium/formoterol 400/12 μg improved trough FEV_1_ vs. placebo at week 24 by 145 mL in ICS users and by 134 mL in non-ICS users (*P* < 0.001 for both comparisons; Fig. 2). In ICS users, aclidinium/formoterol 400/12 µg caused an increase in trough FEV_1_ of 71 mL vs. formoterol alone (*P* < 0.001) and 54 mL (*P* < 0.01) vs. aclidinium alone. A similar pattern of improvement in trough FEV_1_ with aclidinium/formoterol 400/12 µg was observed in non-ICS users compared with formoterol (66 mL; *P* < 0.001), while the difference vs. aclidinium was 14 mL (*P* = 0.356). There were no significant differences in trough FEV_1_ improvements when direct comparisons were made between ICS users and ICS non-users for any treatment group (Table 3).

## Discussion

### Main findings

In this pooled analysis of two pivotal phase III trials, aclidinium/formoterol 400/12 µg twice daily improved bronchodilation in patients with COPD compared with placebo and monotherapies, independent of ICS use. Our findings are reassuring because it would appear that the absence of background ICS use does not blunt the therapeutic response to bronchodilator therapy.

### Interpretation of findings in relation to previously published work

Our results agree with those of the SHINE study, which compared the efficacy of the LABA+LAMA combination glycopyrronium and indacaterol vs. its monocomponents, tiotropium or placebo, and showed that lung function improvements were not influenced by concurrent ICS use.^7^ Furthermore, a recent post-hoc analysis of the OTEMTO studies also showed that the LABA+LAMA combination olodaterol and tiotropium had similar effects on lung function, symptoms and quality of life in patients with COPD using concurrent ICS, compared with non-ICS users.^11^ In the authors’ experience, LABA+LAMA combination inhalers are more likely to be used without concurrent ICS treatment. The current analysis provides further evidence of the magnitude of lung function improvement with a LABA+LAMA combination in patients with COPD not taking ICS.

In the studies reported here, patients using an ICS at baseline were permitted to continue doing so throughout the study, resulting in a proportion of those in the aclidinium/formoterol groups receiving ICS+LAMA+LABA triple therapy. The comparison of triple vs. dual therapy was not an objective of the individual studies or this secondary pooled analysis. However, the fact that some patients received triple therapy provided us with an opportunity to explore and gain further insight into the effect of triple therapy on lung function in COPD. Previously published data demonstrated benefits of triple therapy over dual therapy with an ICS plus a single bronchodilator, and our results also demonstrated similar findings.^12–14^

While in many European countries high proportions (up to 86%) of patients with COPD are treated with ICS,^15–19^ the burden of exacerbations would not support such widespread use. In fact, in a UK primary-care database, only 28% of patients with COPD met the Global Initiative for Chronic Obstructive Lung Disease (GOLD) criteria for high-risk, frequent exacerbators.^20^ The percentage of patients using an ICS at baseline in our analysis ranged from 38.7% to 40.0%. However, our finding that ICS users had higher annual per-patient exacerbation rates vs. non-ICS users would be consistent with a subset population characterised by an exacerbation phenotype.

This post-hoc analysis found that improvements in lung function were similar between ICS users and non-users; however, in a previously published pooled analysis of these studies, differences in exacerbation rate and dyspnoea were observed with concomitant ICS use.^6^

ICS users had higher annual per-patient exacerbation rates vs. non-ICS users: 0.67 vs. 0.36 Healthcare Resource Utilisation (HCRU)-defined exacerbations; aclidinium/formoterol 400/12 µg reduced the risk of moderate/severe HCRU exacerbations vs. placebo in ICS users (rate ratio = 0.56; *P* < 0.05), but not in ICS non-users (rate ratio = 0.83; *P* = 0.47).^6^ It is noteworthy that FEV_1_ was lower in the ICS group at baseline. These observations further underscore that ICS users in these clinical trials represent a subset of patients with different characteristics to ICS non-users; for example, they have more severe airflow obstruction and more exacerbations. Our previous analysis showed that the effect of aclidinium/formoterol 400/12 µg on exacerbations was different in these two groups, but here we show that the effects on lung function are similar.

In our previous pooled analysis, aclidinium/formoterol 400/12 µg caused clinically relevant improvements in dyspnoea (transitional dyspnoea index [TDI] focal score) vs. placebo regardless of concomitant ICS use. Interestingly, there were no significant improvements in TDI focal score for aclidinium/formoterol 400/12 µg vs. monotherapies in patients receiving ICS; however, there were improvements in ICS non-users.^6^ This suggests that, in terms of breathlessness, dual LAMA/LABA therapy has advantages over monotherapies in those patients who are not receiving ICS.

### Strengths and limitations of this study

These are post-hoc analyses, which means that it is difficult to draw definitive conclusions. However, the large sample size means that the data on lung function presented here are likely to be a robust estimate of the effect size. Patients were not randomised by ICS use in the original studies, resulting in the observed differences in airflow obstruction and exacerbation rate between ICS and non-ICS populations at baseline. While this could be considered a limitation of this analysis, it should also be highlighted that if treatment recommendations are being applied consistently, one would expect the two populations to have different characteristics, particularly in terms of exacerbation rate. In order to circumvent these inherent differences at baseline, this analysis focused on the comparison of aclidinium/formoterol vs. placebo and monotherapies within two subgroups with and without concomitant ICS use. However, direct between-group comparisons were also performed and there were found to be no significant differences in lung function end points between ICS users and non-users.

### Implications for future research, policy and practice

The analysis reported here demonstrates that aclidinium/formoterol 400/12 µg improves lung function compared with placebo and monotherapies regardless of ICS use. It is not clear whether this relates to recent research suggesting that lung function is a relatively poor predictor of exacerbations,^21^ but it does suggest that while there is a common benefit of this dual combination bronchodilator on lung function in both groups of patients, this lung-function effect is associated with different clinical benefits depending on whether or not a concomitant ICS is used. It is useful to note that prior to the 2017 update, the GOLD strategy recommended treatment based on risk rather than exacerbation phenotype, allowing most of the patients in GOLD groups C and D to qualify for these categories based on lung function alone, rather than frequent exacerbations, or both.^22^ A relevant clinical message that emerges from our findings is the importance of identifying patients with an exacerbation phenotype who are most likely to benefit from ICS in combination with dual bronchodilator therapy.

## Conclusion

The analysis reported here demonstrates that aclidinium/formoterol 400/12 µg improves lung function compared with placebo and monotherapies regardless of ICS use. However, this lung-function effect is associated with different clinical benefits depending on whether or not a concomitant ICS is used, highlighting the importance of identifying patients who are most likely to benefit from ICS in combination with dual bronchodilator therapy, such as aclidinium/formoterol.

## Methods

This was a subgroup analysis of pooled data from ACLIFORM (NCT01462942) and AUGMENT (NCT01437397) according to concomitant ICS use (user vs. non-user) for aclidinium/formoterol 400/12 μg (the approved dose). Methods, overall efficacy, tolerability and safety results have been published previously.^4, 5^ In brief, the studies included adults aged ≥40 years, current or former smokers (≥10 pack-years) with moderate to severe stable COPD, a post-bronchodilator FEV_1_/forced vital capacity ratio of <70% and a post-bronchodilator FEV_1_ ≥30% and <80% predicted. Patients with a history or current diagnosis of asthma, a respiratory tract infection or COPD exacerbation within 6 weeks (<3 months if hospitalised) prior to screening, or clinically significant respiratory or cardiovascular conditions other than COPD were excluded. Patients were randomised (by centralised interactive voice response system) to receive twice-daily aclidinium/formoterol 400/12 μg, aclidinium/formoterol 400/6 μg, aclidinium 400 μg, formoterol 12 μg or placebo (all via the dry-powder inhaler Genuair™/Pressair^®a^). Concomitant use of ICS, oral or parenteral corticosteroids (≤10 mg/day or 20 mg every other day of prednisone) was allowed if treatment was stable ≥4 weeks prior to screening. The use of albuterol/salbutamol was permitted as rescue medication.

Here, we report results for the co-primary efficacy end points, change from baseline in morning pre-dose (trough) and morning 1-h post-dose FEV_1_ at week 24. Lung function was assessed by standardised spirometric techniques at each study visit (weeks 1, 4, 12, 18 and 24). Trough FEV_1_ was derived as the average of the two best FEV_1_ values obtained before the morning dose, and morning 1-h post-dose FEV_1_ was the maximum FEV_1_ reading from 1 h after the morning dose.

The studies were approved by an independent ethics committee at each site and were conducted in accordance with the Declaration of Helsinki, the International Conference on Harmonisation and Good Clinical Practice. All patients provided written informed consent.

### Statistical analyses

Efficacy analyses were performed in the intent-to-treat population (all randomised patients who took ≥1 dose of study medication and had a baseline and ≥1 post-baseline FEV_1_ assessment). Analysis of the co-primary end points was performed via a mixed model for repeated measures, stratified for concomitant ICS use (prespecified for morning 1-h post-dose FEV_1_ and trough FEV_1_) with treatment group, sex, smoking status, visit, subgroup, and treatment-group-by-subgroup, treatment-group-by-visit and treatment-group-by-visit-by-subgroup interactions as fixed-effect factors, corresponding baseline values and age as covariates, and pre- and post-bronchodilator FEV_1_ as a covariate for FEV_1_ end points. For this pooled evaluation, data analysis was carried out post-hoc and, therefore, no adjustment was made for multiplicity.

^a^Registered trademarks of AstraZeneca PLC; for use within the USA as Pressair^®^ and Genuair^TM^ within all other licensed territories.
